# Trophic ecology of sympatric small cats in the Brazilian Pampa

**DOI:** 10.1371/journal.pone.0201257

**Published:** 2018-07-27

**Authors:** Raissa Prior Migliorini, Felipe Bortolotto Peters, Marina Ochoa Favarini, Carlos Benhur Kasper

**Affiliations:** 1 LABIMAVE (Laboratório de Biologia de Mamíferos e Aves), Universidade Federal do Pampa, São Gabriel, RS, Brazil; 2 Área de vida–Consultoria Ambiental, Canoas, RS, Brazil; Sichuan University, CHINA

## Abstract

Information about resource partitioning among small cat species that live in sympatry in South America is fairly incomplete. Knowledge about feeding habits is essential for understanding the role of these predators in the environment, the impact on prey populations, and potential competition among themselves and with other carnivores. This study aimed to describe and compare the diet of four sympatric small cats in the grasslands of southern Brazil. We analysed the stomach contents of 37 Geoffroy’s cats (*Leopardus geoffroyi*), 27 margays (*Leopardus wiedii*), 14 pampas cats (*Leopardus colocola*), and 20 jaguarundis (*Herpailurus yagouaroundi*) obtained as road kill in the Brazilian Pampa in southern Brazil. Small mammals were the most representative class consumed by all cats, followed by Aves, Reptilia, and Amphibia. Some items, such as rodents *Cavia aperea*, *Akodon* sp., *Oligoryzomys* sp. and Passeriformes were consumed by all cat species. Niche overlap varied widely, from 10% (margay x jaguarundi) to 92% (jaguarundi x pampas cat). Niche breadth indicated that jaguarundi were the most specialized of the cats (*B*_*sta*_ = 0.24) in this region, with a diet closely associated to *C*. *aperea*. Margay consumed more items associated with arboreal behaviour than other cat species, but consumed more terrestrial items than arboreal ones. The pampas cat consumed mostly terrestrial species associated with open fields. Geoffroy’s cat consumed mammals found in a diversity of habitats, indicating high ecological flexibility. Species with more similarity in diet such as jaguarundi and pampas cat probably present temporal segregation in activity. In conclusion, despite their habitat and diet similarities, these four species explore distinct microhabitats by foraging different prey groups, what favor them to live in sympatry.

## Introduction

The order Carnivora comprises mammals adapted to predatory behaviour. Carnivores have an important role in community structure, controlling the abundance of their prey leading to a biological balance in the environment [1]. Among the families of this group, Felidae is clearly monophyletic with a recent diversification of species [2]. Besides the large variability in body size (varying from 1 to 325 kg), body architecture in felids is highly conserved, which could reflect the hypercarnivory habit of this group [3–4]. Therefore, felids make up a good model for understanding how closely related species coexist in the same habitat [5].

The Neotropical region presents a rich diversity of felids with many species living sympatrically. In temperate grasslands of the Brazilian Pampa, the usual felid assemblage is composed of Geoffroy’s cat (*Leopardus geoffroyi* d’Orbigny & Gervais, 1844), pampas cat (*Leopardus colocola* Molina, 1782), margay (*Leopardus wiedii* Schinz, 1821) and jaguarundi (*Herpailurus yagouaroundi* É. Geoffroy Saint-Hilaire, 1803) [6]. This assemblage of species is unique in the world due to the northeastern distribution of Geoffroy’s cat, the southern distribution of the margay and jaguarundi, and the site of occurrence of a disjoint population of pampas cat [7] recognized as subspecies *L*. *colocola munoai* [8]. In the Brazilian Pampa, these species present a high degree of overlap in several ecological aspects, such as in habitat use, activity patterns, size, and food habits, suggesting the existence of interspecific competition [9]. Despite their importance in food webs, mesopredators have received little attention from researchers [10].

Cat species found in Brazilian Pampa have individualized ecological features. All four species are similar in size, varying from 3.3 kg in the margay [11] to 5.2 kg in the jaguarundi [6]. However, jaguarundi is more active at daytime [5] and uses a variety of environments including open fields and forest habitats. The margay seems predominantly nocturnal, and presents a clear association with arboreal activity [11]. The pampas cat is the least known species, but apparently prefers open fields. Geoffroy’s cat is primarily associated with grasslands of southern South America, usually considered the most common cat species in the habitats where it occurs [12].

Knowledge on the feeding ecology of carnivores is essential to understanding their role in the environment, their impact on prey populations, and potential competition among species. In the long run, results from studies focused on diet can be useful for management programs, especially when endangered species are involved [13–14]. Even if they do not disclose details of foraging behaviour, we can infer the predator’s habits from their diet.

The aim of this study was to describe comparatively the food habits of four sympatric small cats in the Brazilian Pampa, to evaluate niche overlap among these species and infer their foraging strategies and habitat preferences. We propose the hypotheses that species with specific habits such margay (arboreal and nocturnal) and jaguarundi (diurnal) will have more discrepant food habits.

## Materials and methods

### Study area

The Brazilian Pampa region occupies a small area in the southern limit of Brazil, representing about 2% of Brazilian territory [15]. This region presents continuity with the grasslands in Uruguay and a small section of the Argentinean province of Entre Ríos, corresponding to the Uruguayan Savannas ecoregion [16], a biome of “tropical and subtropical grasslands, savannas, and shrublands”, defined by Olson et al. [17]. Grass-dominated vegetation types prevail, with sparse shrub and tree formations co-occurring within the grassland matrix. This lies within the South Temperate Zone and has both subtropical and temperate climates with hot summers, cool winters, and no dry season[18], classified as Cfa (humid subtropical) by Köppen’s climatic classification [19].

### Data collection and identification

From October 2013 to August 2017, we opportunistically collected stomach contents of 98 road-killed specimens of four cat species, along highways of Rio Grande do Sul state, Brazil. All dead cats found in the road were exanimate (in the lab or in the field) for the presence of food in the stomach. The stomach contents, when present, were stored in alcohol 70% for diet analysis. Individuals with empty stomachs were not taking into account for this study. Preserved individuals were taken to Laboratório de Biologia de Mamíferos e Aves (LABIMAVE) of Universidade Federal do Pampa (UNIPAMPA), taxidermised and included in the scientific collection of the Institution, as voucher specimens. Samples of muscular tissue were collected from all individuals included in this study, stored in alcohol 96% for future molecular analyses or to solve any doubts in the identification of individuals.

The macroscopic items contained in the stomachs such as hairs, teeth, feathers, beaks, and scales were separated and identified to the lowest possible taxonomic level of prey consumed by these cats. Mammal prey was mainly identified by microscopic hair patterns and bird feathers based on barbule nodules of the down [20], comparing with the reference collection of LABIMAVE. Reptiles and amphibians were identified by consulting a specialist.

### Diet analysis

Food items were expressed in terms of Frequency of Occurrence (FO), where the number of samples in which one prey type occurred divided by the total number of samples multiplied by 100, and Percentage of Occurrence (PO), calculated by dividing the number of occurrences of a prey type by the total occurrences of all prey types multiplied by 100. The FO indicates how common an item was in the diet, while the PO indicates the relative importance of an item in the diet.

Similarity among diets was evaluated using the clustering technique with the unweighted pair-group method and arithmetic averaging (UPGMA) based on the proportion of each prey type using the Morisita’s index on software PAST 2.17c [21].

Diet overlap between species pairs were calculated using Pianka’s index [22]: *O*_*jk*_ = Σ*P*_*ij*_*P*_*ik***/**_√Σ*P*_*ij*_^*2*^Σ*P*_*ik*_^*2*^, where *P*_*ij*_ is the proportion of a prey type in one predator’s diet and *P*_*ik*_ is the proportion of the same prey type in a second predator’s diet. The index ranges from 0 (no food resources in common) to 1 (total overlap of food niches). The proportion of each prey type was calculated through their relative volume estimated on a nine-point scale: 0 (absence), 1 (< 1%), 2 (1–5%), 3 (6–10%), 4 (11–25%), 5 (26–50%), 6 (51–75%), 7 (76–98%), and 8 (> 98%). For the niche overlap calculation, scores were converted to the midpoint of each percentage interval (1 = 0.5%, 2 = 3%, 3 = 8%, 4 = 18%, 5 = 38%, 6 = 63%, 7 = 87%, 8 = 99%), following Kruuk & Parish [23], Ray & Sunquist [24], and Kasper et al. [25].

To evaluate trophic niche breadth we used Levins’ index: *B* = 1 **/** (Σ*p*^*2*^j), where *p*j is the Percentage of Occurrence of a prey type [26]. This index was standardized to a scale ranging from 0 (generalist habit, when prey items are consumed in equal proportions) to 1 (specialized diet, when few prey categories are eaten in greater frequency, while most are eaten in lower frequency) [27]: *B*_*sta*_ = (*B*-1) **/** (*n*-1), were *B* is Levins’ index and *n* is the total number of prey types consumed.

Major axes of dietary variation among the small cat species were identified through a correspondence analysis on software PAST 2.17c [21]. Only the proportions of prey types that composed at least 5% of the diet were included in this analysis. The average adult body mass and primary lifestyle of mammal prey were obtained from the literature [28–31]. We estimated the minimum number of individuals consumed (MNI) by counting teeth, feet, and tails. When only hairs were encountered, we assumed the MNI equal to 1.

The Index of Relative Importance (*IRI*) combines the frequency, number of individuals, and volume measures into a single estimate of the relative importance of food types [32]: *IRI = F(N + V)*, where *F* is the Frequency of Occurrence, *N* is the Percentage of Occurrence, and *V* is the volumetric percentage. Only *IRI* for mammal prey were considered as this was the most represented group in the diet of the four cats. The volumetric percentage was calculated based on ingested biomass. Small-sized cats can consume 60–90 g per kg of body mass per day [33]. Based on the fresh body weight of individuals collected in this study, we considered the average body mass to be 3.69 kg (SD 0.82) for Geoffroy’s cat (n = 20) and 2.64 kg (SD 0.38) for margay (n = 8); based on the literature we used 3.5 kg for pampas cat, and 5.2 kg for jaguarundi [6]. Considering an average of 75 g of prey consumed daily for each kg of predator, we estimated that cats consumed 276.8, 198, 262.5, and 390 g per day, respectively. These values were used as the estimated ingested biomass of prey too large to be consumed entirely. For prey types with biomass below these values, the ingested biomass was estimated by the multiplication of their average body mass by their MNI.

Taxonomy follows Gardner [34] for marsupials, Patton, Pardinas & D’Elía [35] for rodents, Kitchener et al. [8] for the cats, Bencke et al. [36] for birds, Costa & Bérnils [37] for reptiles and Segalla et al. [38] for amphibians.

## Results

We found 37 prey items altogether in the diet of the four small cats. Mammals were the most frequent prey type, represented mostly by rodents, followed by birds. Reptiles and amphibians were consumed secondary by Geoffroy’s cat and margay, but were not present in the diet of pampas cat and jaguarundi respectively (Table 1, S1 Table).

**Table 1:** Prey items recorded in the stomach contents of four sympatric small cat species in Brazilian Pampa, represented as Frequency of Occurrence (FO); Percentage of Occurrence (PO) and proportional volume (%Vol.).

Items	*Leopardus geoffroyi* (37)			*Leopardus wiedii* (27)			*Leopardus colocola* (14)			*Herpailurus yagouaroundi* (20)		
	FO	PO	%Vol	FO	PO	%Vol	FO	PO	%Vol	FO	PO	%Vol
Mammalia	86.5	65.3	76.5	77.8	52.6	64.7	85.7	63.2	66.2	90.0	66.7	77.4
Didelphimorphia												
Didelphidae												
*Cryptonanus* sp.	2.7	1.4	0.1	7.4	3.9	6.0	-	-	-	-	-	-
*Monodelphis dimidiata*	5.4	2.7	1.1	-	-	-	-	-	-	-	-	-
Rodentia												
Cricetidae												
*Akodon* sp.	5.4	2.7	1.5	11.1	5.8	8.3	7.1	4.8	2.7	5.0	3.4	4.4
*Deltamys kempi*	8.1	4.1	3.2	3.7	1.9	0.7	7.1	4.8	0.6	-	-	-
*Holochilus vulpinus*	13.5	6.8	6.9	3.7	1.9	1.4	-	-	-	-	-	-
*Oxymycterus nasutus*	18.9	9.5	6.1	-	-	-	-	-	-	-	-	-
*Oligoryzomys* sp.	27.0	13.5	8.9	33.3	17.3	19.8	28.6	19.1	17.4	10.0	6.9	4.1
*Wilfredomys oenax*	-	-	-	14.8	7.7	9.5	-	-	-	-	-	-
Unidentified Cricetidae	2.7	1.4	1.0	14.8	7.7	9.0	-	-	-	5.0	3.3	0.9
Muridae												
*Mus musculus*	8.1	4.1	5.4	-	-	-	-	-	-	-	-	-
*Rattus norvegicus*	5.4	2.7	2.7	3.7	1.9	3.2	-	-	-	-	-	-
*Rattus rattus*	8.1	4.1	4.4	11.1	5.8	7.6	-	-	-	-	-	-
Caviidae												
*Cavia aperea*	35.1	17.6	24.2	3.7	1.9	0.3	50.0	33.3	46.1	70.0	48.3	68.1
Dasyproctidae												
*Dasyprocta azarae*	8.1	4.1	7.4	-	-	-	-	-	-	-	-	-
Lagomorpha												
Leporidae												
*Lepus europaeus*	5.4	2.7	5.3	-	-	-	-	-	-	5.0	3.4	0.4
Aves	29.7	22.5	12.9	55.6	37.5	26.5	42.9	31.6	27.8	25.0	18.5	12.3
Tinamiformes	2.7	1.4	0.2	-	-	-	14.3	9.5	5.4	-	-	-
Anseriformes	-	-	-	-	-	-	-	-	-	5.0	3.4	1.9
Galliformes	2.7	1.4	2.7	-	-	-	-	-	-	-	-	-
Pelecaniformes	-	-	-	3.7	1.9	3.7	-	-	-	5.0	3.4	4.9
Gruiformes	8.1	4.1	3.1	-	-	-	-	-	-	-	-	-
Columbiformes	5.4	2.7	1.1	22.2	11.5	8.9	-	-	-	5.0	3.4	0.9
Psittaciformes	-	-	-	-	-	-	7.1	4.8	4.5	-	-	-
Cuculiformes	-	-	-	7.4	3.9	1.5	7.1	4.8	7.1	-	-	-
Passeriformes	8.1	4.05	2.3	25.9	13.5	11.8	21.4	14.3	10.0	15.0	10.3	4.5
Unidentified Aves	2.7	1.35	1.0	11.1	5.7	0.9	-	-	-	5.0	3.4	0.2
Reptilia	10.8	8.16	5.6	7.4	5.0	6.0	-	-	-	20.0	14.8	8.6
Squamata												
Anguidae												
*Ophiodes* sp.	-	-	-	-	-	-	-	-	-	5.0	3.4	0.4
Teiidae												
*Salvator merianae*	-	-	-	3.7	1.9	2.3	-	-	-	5.0	3.4	1.9
Dipsadidae												
*Atractus reticulatus*	2.7	1.4	0.2	-	-	-	-	-	-	-	-	-
*Erythrolamprus poecilogyrus*	2.7	1.4	2.7	-	-	-	-	-	-	-	-	-
*Thamnodynastes hypoconia*	-	-	-	3.7	1.9	3.7	-	-	-	-	-	-
*Thamnodynastes strigatus*	-	-	-	-	-	-	-	-	-	5.0	3.4	4.4
Elapidae												
*Micrurus altirostris*	-	-	-	-	-	-	-	-	-	5.0	3.4	1.9
Viperidae												
*Bothrops pubescens*	2.7	1.4	1.0	-	-	-	-	-	-	-	-	-
Unidentified Squamata	2.7	1.4	1.7	-	-	-	-	-	-	-	-	-
Amphibia	5.4	4.1	3.7	7.4	5.0	0.4	7.1	5.3	4.5	-	-	-
Anura												
Leptodactylidae												
*Leptodactylus chaquensis*	-	-	-	-	-	-	7.1	4.8	4.5	-	-	-
Unidentified Anura	5.4	2.70	3.7	3.7	1.9	0.1	-	-	-	-	-	-
Unidentified Hylidae	-	-	-	3.7	1.9	0.1	-	-	-	-	-	-

Plant material, represented by leaves of grass (Poaceae), and invertebrates were sporadically ingested by all four cats. In our analyses, we excluded these items because they could be accidentally consumed (insects) or just for digestive purposes (grass). Moreover these items represent a minimal part of ingested biomass in the diet.

Comparatively, the niche breadth based on prey identified to class level showed Geoffroy’s cat as the most specialized (*B*_*sta*_ = 0.35), and pampas cat and jaguarundi as the most generalist (*B*_*sta*_ = 0.50). Refining the analysis with the POs of prey identified to specific level, jaguarundi was the most specialized (*B*_*sta*_ = 0.24) and margay was the most generalist (*B*_*sta*_ = 0.56) (Table 2). Based on the relative volume of type of prey identified to class level, niche overlap was high for all pairs of species (96–99%), but decreased when prey was identified to species level (10–92%) (Table 2).

**Table 2:** Standardized niche breadths (*B*_*sta*_) and food niche overlap between pairs of four sympatric small cat species in the Brazilian Pampa. Values to the left and below the x diagonal and *B*_*sta*_ were calculated based on the lowest level of prey classification. Values to the right and above are based on broader prey classification (*B*_*sta*_*’*).

	*L*. *geoffroyi*	*L*. *wiedii*	*L*. *colocola*	*H*. *yagouaroundi*	*B*_*sta*_	*B*_*sta*_*’*
*L*. *geoffroyi*	x	0.97	0.97	0.99	0.49	0.35
*L*. *wiedii*	0.31	x	0.99	0.97	0.56	0.46
*L*. *colocola*	0.82	0.31	x	0.96	0.54	0.50
*H*. *yagouaroundi*	0.80	0.10	0.92	x	0.24	0.50

**Fig 1:**
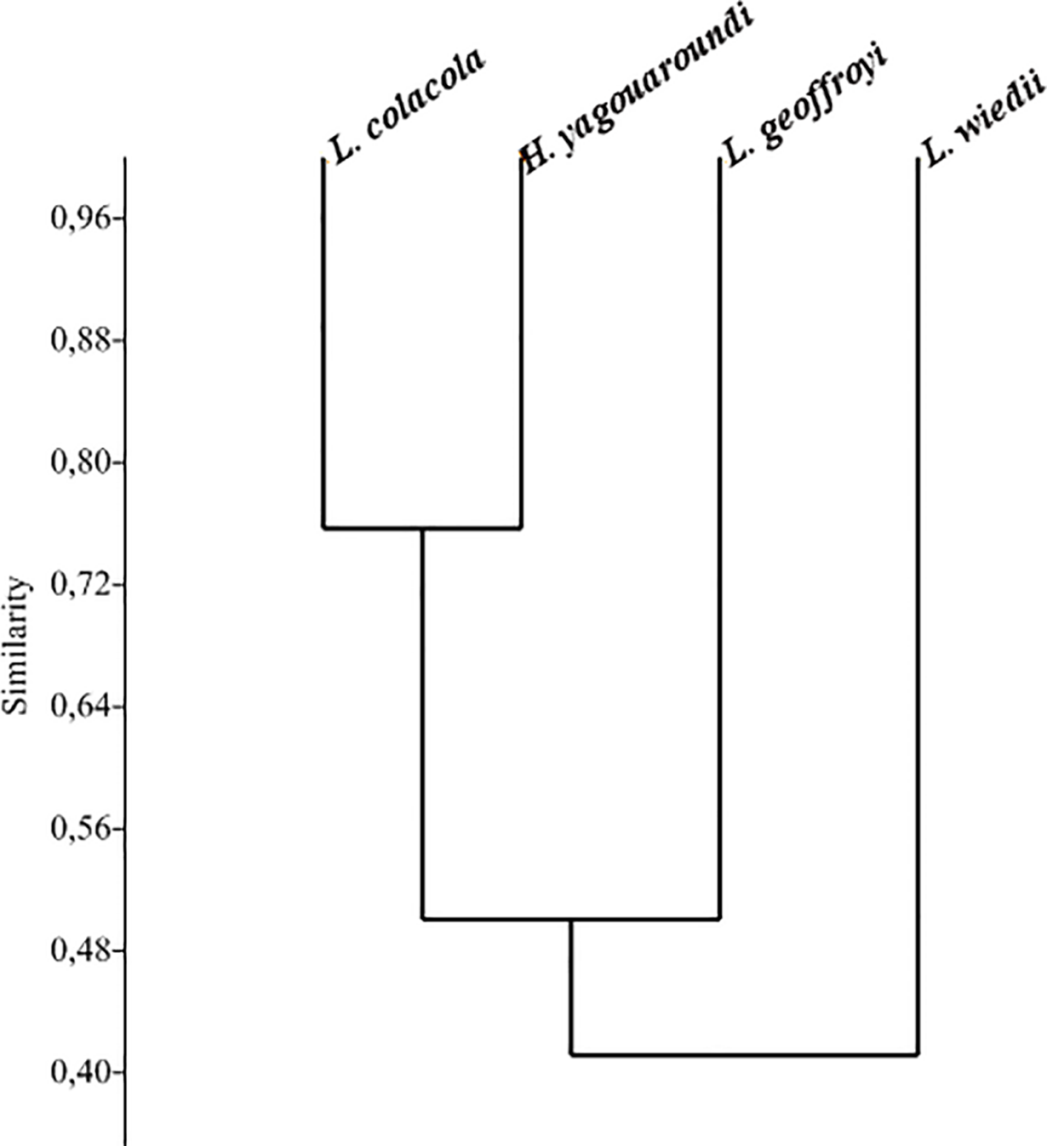
Similarity (Morisita’s index) in the diet of *Leopardus geoffroyi*, *Leopardus wiedii*, *Leopardus colocola*, and *Herpailurus yagouaroundi* in the Brazilian Pampa based on the proportional volume estimated by eye of the items in the diet.

**Fig 2:**
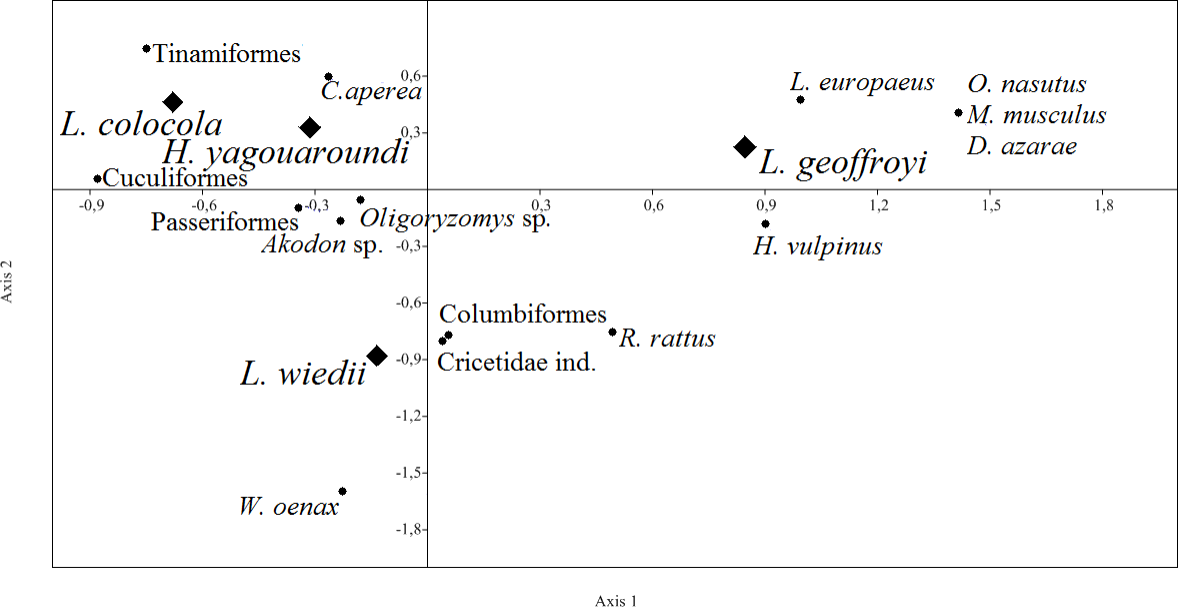
Correspondence analysis of the main food groups (circles) in the diet of four sympatric small cats (diamonds) in the Brazilian Pampa. The first axis accounts for 45.5% of the total variation and the second accounts for 40.2%.

**Fig 3:**
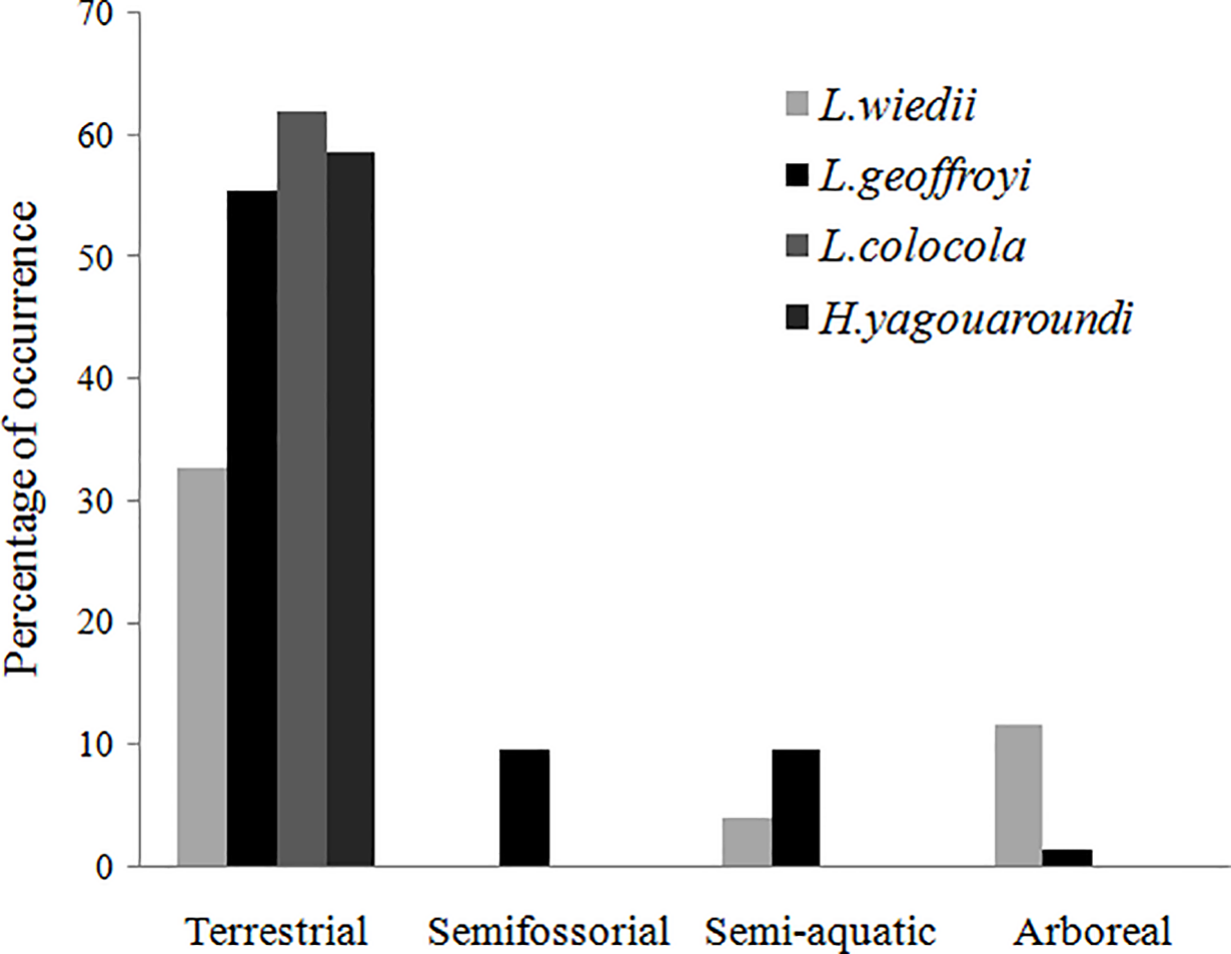
Primary lifestyle of mammalian prey identified in *L*. *geoffroyi*, *L*. *wiedii*, *L*. *colocola*, and *H*. *yagouaroundi* stomach contents from Brazilian Pampa, based on the Percentage of Occurrence of prey items.

The dendrogram of similarity grouped pampas cats and jaguarundi as having the most similar diets while margay showed the least similarity with the other species (Fig 1). The first two axis of correspondence analyses accounted for 85.7% of the total variation in the diet of the four cats (Fig 2). Although present in the diet of all species, the Brazilian guinea pig, *Cavia aperea*, showed a greater association with pampas cats and jaguarundi. Terrestrial mammals occurred in the diet of all four cats, comprising up to 61.9% and 58.6% of the diet of the pampas cat and jaguarundi, respectively. The semifossorial *Oxymycterus nasutus* was consumed only by Geoffroy’s cat. Semi-aquatic *Holochilus vulpinus* and *Rattus norvegicus* and the arboreal *Cryptonanus* sp. and *Wilfredomys oenax* were consumed both by Geoffroy’s cats and margay (Table 3 and Fig 3).

**Table 3:** Mammalian prey species’ contribution to the diet of four small sympatric cats in Brazilian Pampa.

Mammalian prey	Average body mass (g)	Primary lifestyle	*Leopardus geoffroyi*		*Leopardus wiedii*		*Leopardus colocola*		*Herpailurus yagouaroundi*	
			MNI	*IRI*	MNI	*IRI*	MNI	*IRI*	MNI	*IRI*
*Cryptonanus* sp.*	17.0^[^^31^^]^	Arb^[^^31^^]^	1	6.2	3	187.8	-	-	-	-
*Monodelphis dimidiata*	62.0^[^^31^^]^	Ter^[^^31^^]^	2	41.5	-	-	-	-	-	-
*Akodon* sp.*	32.0^[^^31^^]^	Ter^[^^28^^]^	2	30.8	3	321.8	1	133.5	1	67.3
*Deltamys kempi*	26.0^[^^31^^]^	Ter^[^^28^^]^	3	96.2	1	38.8	1	70.4	-	-
*Holochilus vulpinus*	210.0^[^^31^^]^	SeA^[^^31^^]^	5	331.8	1	49.9	-	-	-	-
*Oxymycterus nasutus*	50.0^[^^31^^]^	SeF^[^^31^^]^	9	435.0	-	-	-	-	-	-
*Oligoryzomys* sp.*	22.5^[^^31^^]^	Ter^[^^28^^,^^30^^]^	10	783.7	10	3245.8	5	2136.7	4	269.1
*Wilfredomys oenax*	75.0^[^^31^^]^	Arb^[^^31^^]^	-	-	13	827.7	-	-	-	-
*Mus musculus*	25.5^[^^29^^]^	Ter^[^^29^^]^	7	119.1	-	-	-	-	-	-
*Rattus norvegicus*	325.0^[^^29^^]^	SeA^[^^28^^]^	2	48.3	1	49.9	-	-	-	-
*Rattus rattus*	140.0^[^^29^^]^	Ter^[^^28^^]^	4	110.1	3	465.0	-	-	-	-
*Cavia aperea*	549.0^[^^31^^]^	Ter^[^^28^^,^^31^^]^	13	2438.8	1	48.8	7	6499.9	17	12945.0
*Dasyprocta azarae*	2900.0^[^^31^^]^	Ter^[^^31^^]^	3	132.4	-	-	-	-	-	-
*Lepus europaeus*	4750.0^[^^30^^]^	Ter^[^^30^^]^	2	63.1	-	-	-	-	1	49.9

Arb, arboreal; Ter, terrestrial; SeA, semi-aquatic; SeF, semifossorial; MNI, Minimum Number of Individuals; *IRI*, Index of Relative Importance.

*average body mass calculated based on the average adult body mass of all the species in the genus occurring in Brazilian Pampa.

## Discussion

The general pattern found in the diet of small cats from Brazilian Pampa corresponds to that expected for felids, being mammals the predominant group [39]. Small mammals are profitable prey for carnivores due to their high abundance in the ecosystem and a higher percentage of digestible biomass in relation to reptiles and birds of the same size [40].

Previous literature has reported great variation in niche breadth throughout the species distribution [25, 41–51]. However, we must be careful when comparing these results as the level of prey taxonomic resolution varied greatly. In general, classification to species level give larger niche breadths, while a broader level of prey classification results in lower values [52] as observed here occurring with Geoffroy’s cat, margay, and pampas cat (Table 2). In this work, margay was found to be the most generalist cat (*B*_*sta*_ = 0.56) despite Geoffroy’s cat preying on a higher diversity of taxa. This apparently paradoxical result occurs because margay consumes its prey more equitably than Geoffroy’s cat, which consumes some items more intensively while others just occasionally.

As expected, the largest discrepancy in diets was found between margay and jaguarundi, with only 10% overlap in food niches. These cats ate seven prey items in common, but in highly different proportions. They showed the high difference in the consumption of Brazilian guinea pigs (3.7% in margay samples versus 70% in jaguarundi’s). As margays have mostly arboreal habits, this cat could explore more microhabitats than the jaguarundi, despite their functionally identical craniomandibular characteristics [53]. Margay’s arboreal ability may allow them to take advantage of catching prey in the upper strata, providing opportunities for greater diversification in niche occupancy [54]. Our results reinforce the idea that their competition is reduced throughout temporal and spatial partitioning [5], reflected by the margay including more arboreal prey in its diet (Fig 3).

The Brazilian guinea pig was consumed by all cat species, which could be related to its habits, wide distribution, and tolerance to environmental disturbances. It is frequently found in linear habitats, as on margins of roadsides, with a zone of tall and dense vegetation that provides protection and a more open zone of short vegetation for foraging [55]. Thus, not only is the landscape of our study area compatible with its suitable habitat, but the road favours its presence and the road-killed cats. Because Brazilian guinea pigs are crepuscular [29], their peak of activity overlaps with the end and beginning of the activity of both the three nocturnal cats and the diurnal jaguarundi [5, 56].

The other two mammals consumed by all cats, *Akodon* sp. and *Oligoryzomys* sp. are represented by two species for each genus in Brazilian Pampa [57]. Through our collections in this region, we have found both genera to be the most common and abundant rodent in different habitats (*unpublished data*). Passeriformes corresponds to the most diversified order of Aves, comprising almost 50% of bird species in southern Brazil [36]. Its high species diversity, habits, and lifestyles may explain why this was the only bird order consumed in common by all cats.

Regarding the ecological characteristics of mammal prey, Geoffroy’s cat was the only species that consumed small mammals found in all vertical strata considered here (arboreal, terrestrial, semiaquatic, and fossorial). This indicates its great trophic flexibility, as it seems to be able to adjust its feeding behavior according to availability or vulnerability of prey [43, 58–60]. This behavior could explain why this is the most common small cat in Brazilian Pampa. This is important for species’ survivorship, as the intensification of livestock and agricultural activities has led to an increase in areas of cultivated pastures and a decrease in natural fields [18].

Additionally, in the margay’s stomach contents, young individuals of Columbiformes and Cuculiformes were found, which indicates predation in nests from arboreal stratum. Considering only mammal prey, terrestrial rodents were more important (total *IRI* of 4120.2) than arboreal ones (total *IRI* of 1015.5) (Table 3). This corroborates Kasper, Schneider & Oliveira [61], who suggested that this cat is active mostly on the ground at night and uses trees during the day as resting sites, hunting opportunistically in this stratum.

Data about pampas cats showed here are the first for the subspecies *L*. *colocola munoai*. The Brazilian guinea pig, the most important small mammal in its diet, inhabits open fields, swamp edges, and roadside habitats [55, 57]. In Pampa, these habitats are typical of other important prey in its diet, such as rodents of the genus *Oligoryzomys* [30, 57] and the most common Tinamiformes birds [62]. Based on prey habits, it is possible to infer that pampas cats prefer open fields with shrub cover and the edges of swamps for foraging terrestrial prey in the savannas of Brazilian Pampa.

The specificity of jaguarundi (*B*_*sta*_ = 0.24) consuming only three mammalian taxa was unexpected due to the great diversity of prey described in the literature. The low diversity of small mammals in Brazilian Pampa in relation to Atlantic Forest, where most cat studies have been conducted [41–42, 47, 49, 51, 63–64], could explain the low prey diversity in the jaguarundi’s diet found here. In this temperate grassland, the cat’s diet was based mainly on the consumption of Brazilian guinea pigs (Table 3).

Most human-felid conflicts in the Brazilian Pampa are related to poultry predation. The cats are captured and killed in retaliation near rural residences [65]. However, we observed only two occurrences of domestic animal depredation by cats. Galliformes (chickens) represented only 1.35% of the Geoffroy’s cat diet whereas Anseriformes (domestic goose) composed only 3.4% of the jaguarundi’s diet. The impact of small cats on the economy of small producers seems negligible and certainly does not justify the preventive killing of these animals, which is cited as a threat to small cats [12, 66].

In this study we found a partial overlap in the use of resources, as some items were consumed exclusively by one cat species, while others were consumed by all. The small cats in Brazilian Pampa may have evolved to adopt different foraging strategies to decrease competition. It is interesting to note that these small cats no longer suffer from the influence of top predators that dominated this system before colonization [12] in the XVII and XVIII centuries. As a result, what we see today might be a reflection of a new arrangement of resource partitioning among these mesocarnivores.

## Supporting information

S1 Table Prey items recorded in the stomach contents of four sympatric small cat species in Brazilian Pampa. (XLSX)
